# Correction: CFD study on microchannel reactor operating conditions for Fischer–Tropsch synthesis with Fe-based catalysts

**DOI:** 10.1039/d6ra90004f

**Published:** 2026-01-19

**Authors:** Shijie Ren, Yuanyang Wang

**Affiliations:** a Taiyuan University of Science and Technology, College of Materials Science and Engineering, College of Energy and Materials Engineering 66 Waliu Road Wanbailin District Taiyuan Shanxi Province 030024 China; b Taiyuan University of Science and Technology, College of Chemical Engineering and Technology 66 Waliu Road Wanbailin District Taiyuan Shanxi Province 030024 China yywangs@163.com

## Abstract

Correction for ‘CFD study on microchannel reactor operating conditions for Fischer–Tropsch synthesis with Fe-based catalysts’ by Shijie Ren *et al.*, *RSC Adv.*, 2025, **15**, 13137–13151, https://doi.org/10.1039/D5RA01314C.

The authors regret that the affiliations (*a* and *b*) were incorrectly shown in the original manuscript. A corrected version of the affiliation list, in which the Taiyuan University of Technology has been corrected to Taiyuan University of Science and Technology, is shown here. The Royal Society of Chemistry apologises for these errors and any consequent inconvenience to authors and readers.

